# Differences in arithmetic performance between Chinese and German adults are accompanied by differences in processing of non-symbolic numerical magnitude

**DOI:** 10.1371/journal.pone.0174991

**Published:** 2017-04-06

**Authors:** Jan Lonnemann, Su Li, Pei Zhao, Peng Li, Janosch Linkersdörfer, Sven Lindberg, Marcus Hasselhorn, Song Yan

**Affiliations:** 1 Department of Education and Human Development, German Institute for International Educational Research (DIPF), Frankfurt am Main, Germany; 2 Center for Individual Development and Adaptive Education of Children at Risk, Frankfurt am Main, Germany; 3 Key Lab of Behavioral Science, Institute for Psychology, Chinese Academy of Sciences, Beijing, China; 4 Psychology Department, School of Education & Management, Yunnan Normal University, Kunming, China; 5 Paderborn University, Faculty of Arts and Humanities, Paderborn, Germany; 6 Department of Educational Psychology, Institute for Psychology, Goethe-University, Frankfurt am Main, Germany; 7 Department of Psychology & Methods, Jacobs University, Bremen, Germany; Universiteit Utrecht, NETHERLANDS

## Abstract

Human beings are assumed to possess an approximate number system (ANS) dedicated to extracting and representing approximate numerical magnitude information. The ANS is assumed to be fundamental to arithmetic learning and has been shown to be associated with arithmetic performance. It is, however, still a matter of debate whether better arithmetic skills are reflected in the ANS. To address this issue, Chinese and German adults were compared regarding their performance in simple arithmetic tasks and in a non-symbolic numerical magnitude comparison task. Chinese participants showed a better performance in solving simple arithmetic tasks and faster reaction times in the non-symbolic numerical magnitude comparison task without making more errors than their German peers. These differences in performance could not be ascribed to differences in general cognitive abilities. Better arithmetic skills were thus found to be accompanied by a higher speed of retrieving non-symbolic numerical magnitude knowledge but not by a higher precision of non-symbolic numerical magnitude representations. The group difference in the speed of retrieving non-symbolic numerical magnitude knowledge was fully mediated by the performance in arithmetic tasks, suggesting that arithmetic skills shape non-symbolic numerical magnitude processing skills.

## Introduction

Human beings are assumed to possess an evolutionary ancient, innate system dedicated to extracting and representing approximate numerical magnitude information. This system is called the approximate number system (ANS; see [1], 2010, for an overview) and enables us to discriminate between sets of different numerical quantities, a crucial ability for everyday life. We are faster and more accurate in comparing two dot arrays with respect to their quantity the more the ratio deviates from one (e.g., [2]). The ability to discriminate between sets of different numerical quantities has been observed in preverbal infants (e.g., [3]), and it undergoes a progressive refinement throughout development peaking at approximately the age of 30 years [1,4]. The factors underlying this developmental progression are a matter of ongoing debate. While the initial increase in the precision of the ANS probably reflects intrinsic maturational and sensory factors, the further development of the ANS is assumed to be associated with the development of mathematical skills (e.g., [5]).

Recent meta-analyses have lend support to this notion by showing a significant association between non-symbolic numerical magnitude processing skills and symbolic math performance [6,7,8]. In the included studies, the ANS was assessed in non-symbolic numerical magnitude comparison tasks and indexed by different measures like overall accuracy/error rate (ER), overall reaction time (RT), or the internal Weber fraction (*w*), which measures the smallest numerical difference that can be reliably detected on the basis of ER. Chen and Li [6] focused on overall accuracy/ER or *w* and found a significant association with math performance that did not differ significantly between children and adults. On the other hand, Fazio et al. [7] reported higher correlations for overall accuracy/ER or *w* compared to overall RT as well as higher correlations for children compared to adults. Similarly, Schneider et al. [8] detected higher correlations for overall accuracy/ER compared to overall RT and a small moderating effect of age. Chen and Li [6] showed that the association between overall accuracy/ER or *w* and math performance remains significant after considering potential moderators like general cognitive abilities. They, however, also pointed out that other possible confounding variables are worth examining in future studies. In this regard, it could be demonstrated that performance in non-symbolic numerical magnitude comparison tasks depends on the ability to integrate different visual cues and it has recently been suggested that the association between non-symbolic numerical magnitude processing skills and math performance might be mediated by the ability to combine different sensory cues ([9], see also [10]).

Based on findings from longitudinal studies, Chen and Li [6] report that while non-symbolic numerical magnitude processing skills prospectively predict later math performance, they can also be retrospectively predicted by earlier math performance. Thus, mathematical skills seem to shape the ANS and contribute to its developmental progression. According to Chen and Li [6], the estimated effect sizes may however be inaccurate because of the small number of longitudinal studies (six studies with prospective and four studies with retrospective data).

Other methodological approaches to examining the influence of mathematical skills on the ANS have not revealed conclusive results, either. Three studies examined whether schooling has an impact on non-symbolic numerical magnitude processing skills. Zebian and Ansari [11] compared Syrian adults who had attended no more than one year of schooling with Syrian adults who had attended school for more than 10 years. While the two groups did not differ with respect to ER in a non-symbolic numerical magnitude task, literate participants answered significantly faster than illiterate participants. According to the authors, this difference in RT was likely a result of illiterate participants' unfamiliarity with speeded computerized tasks, and does not reflect a group difference in non-symbolic numerical magnitude processing skills. Nys, Ventura, Fernandes, Querido, and Leybaert [12] assessed the ability to discriminate between different numerical quantities in Portuguese adults who had never received math education, in Portuguese adults who had not attended regular school but received math education in adulthood, and in Portuguese adults who had regularly attended school in childhood. Their results revealed that adults who had not been exposed to schooling answered slower and made more errors than members of the other two groups. In a similar vein, Piazza et al. [5] examined two groups of Amazonian Indians, the Mundurucú, one of which had had access to mathematics education, while the other one had not. The two groups did not differ with regard to RT in a non-symbolic numerical magnitude task but those who had been introduced to the concepts of exact symbolic number and arithmetic showed a smaller average *w* (i.e., a better performance).

Another three studies investigated the impact of higher math education on the ANS. Castronovo and Göbel [13] compared psychology and mathematics students with regard to their ability to discriminate between different numerical quantities and their mathematical achievement. While the mathematics students exhibited higher mathematics achievement, they did not perform better in the non-symbolic numerical discrimination task. The authors thus conclude that an extended education in mathematics is not reflected in the ANS. Similarly, Guillaume, Nys, Mussolin, and Content [14] compared psychology and engineering students. In contrast to the findings by Castronovo and Göbel [13], results revealed improved non-symbolic numerical magnitude processing skills (smaller average *w* and similar RT) in the adults with higher mathematical skills, i.e., in engineering students. Recently, Lindskog, Winman, and Juslin [15] compared students majoring in subjects with varying degrees of mathematics (mathematics, business, and humanities) and observed a non-significant trend with participants from more mathematics-oriented courses showing better non-symbolic numerical magnitude processing skills (smaller average *w*).

Taken together, the question of whether better mathematical skills are reflected in non-symbolic numerical magnitude processing skills has not yet been answered fully. In the present study, we thus probed this question by comparing Chinese and German adults with regard to their performance in simple arithmetic tasks and in a non-symbolic numerical magnitude comparison task. Cross-national assessments of mathematical achievement have repeatedly demonstrated that Chinese children outperform their non-Chinese peers (e.g., [16,17,18,19]). Similarly, several studies reported a substantive advantage of young Chinese adults over their non-Chinese peers in simple arithmetic tasks (e.g., [20,21]). Thus, if better arithmetic skills are reflected in non-symbolic numerical magnitude processing skills, a superior Chinese performance should not only exist for arithmetic skills but also for non-symbolic numerical magnitude processing skills. Moreover, if arithmetic skills shape non-symbolic numerical magnitude processing skills, a performance difference between Chinese and German adults in non-symbolic numerical magnitude processing should be mediated by arithmetic skills. Our findings reveal that Chinese participants not only show a higher fluency in solving simple arithmetic tasks but are also able to discriminate between sets of different numerical quantities at a faster pace than their German peers. This group difference in non-symbolic numerical magnitude processing was fully mediated by the performance in arithmetic tasks, suggesting that arithmetic skills shape non-symbolic numerical magnitude processing skills.

## Materials and method

### Participants

Seventy Chinese (34 female, mean age 20.8 [SD 1.6, range 18–25] years) and seventy German university students (34 female, mean age 20.5 [SD 1.5, range 18–25] years) participated in this study. All Chinese participants were Chinese native speakers tested in China, and all German participants were German native speakers tested in Germany. While oral informed consent was obtained from all participants, our study was not approved by an ethics committee. This is due to the fact that data acquisition for our study started in 2011. At the time, it was not common practice to apply for an ethics committee approval for psychological studies involving only cognitive measures like ours.

### Procedure

A non-symbolic numerical magnitude comparison task was used to assess the ANS and arithmetic skills were examined by sets of addition and subtraction problems. To assure that possible between-group differences could not be explained by differences in more general performance factors, reasoning abilities and processing speed were also assessed. Reasoning abilities were examined by Raven’s Standard Progressive Matrices Plus (SPM Plus; [22]). All participants started with the non-symbolic numerical magnitude comparison task, then proceeded with the arithmetic tasks, and finally worked on the task assessing reasoning abilities. The different tasks were carried out individually. In a subgroup of participants (50 Chinese and 50 German participants), processing speed was assessed by a visual detection task. This task was carried out after the non-symbolic numerical magnitude comparison task.

#### Non-symbolic numerical magnitude comparison

Sets of black dots were presented in two white circles on the left and the right hand side of the screen of a computer running Presentation^®^ software (Neurobehavioral Systems, Inc.). From a viewing distance of about 60 cm, each of the white circles had a visual angle of 9.91^°^ (104 mm) and the black dots ranged between .48 and .95^°^ (5–10 mm). On each trial, one of the white circles contained either 16 or 32 dots (reference numerosities) and the other one contained between 12 and 20 dots (deviants) for the 16 dot reference and between 24 and 40 dots for the 32 dot reference. See Table 1 for a depiction of the different comparison pairs. Each of the 16 comparison pairs appeared eight times, four times with the reference numerosity on the left and four times on the right hand side. Every single comparison pair had a unique configuration of dots. In half the 16 trials per comparison pair, the size of the area occupied by the dots in each circle was held constant (luminance-controlled trials), while in the other half, individual dot size in each circle was held constant (size-controlled trials). Participants were asked to indicate, without using counting strategies, the side of the larger numerical magnitude by answering with the left index finger when it was larger on the left hand side and by using the right index finger when it was larger on the right hand side. Responses were given by pressing the left and right CTRL-buttons of the computer’s keyboard. Reaction times (RT) and errors (ER) were recorded, and the instruction stressed both speed and accuracy. The order of trials was pseudo-randomized so that there were no consecutive identical comparison pairs. The experiment started with eight warm-up trials (data not recorded), followed by in total 128 experimental trials (16 comparison pairs × 2 perceptual control conditions × 4 repetitions). A trial started with the presentation of a black screen for 700 ms. After the black screen had vanished, the target appeared until a response was given, but only up to a maximum duration of 4000 ms. If no response was given, a trial was classified as erroneous. No feedback was given regarding the correctness of responses. Mean RT and mean ER were used as individual markers of the ANS (see e.g., [23], for a discussion on different indices of the ANS). In order to look for possible differences between luminance-controlled and size-controlled trials in the non-symbolic numerical magnitude comparison task, we also computed mean RT and mean ER for both conditions separately. Correct responses were used for computing mean RT. Response times below 200 ms were excluded from further analysis. This trimming resulted in .00% of response exclusions for Chinese participants and in .03% of response exclusions for German participants. Response times were log-transformed to yield more normally distributed data (the Shapiro-Wilk test revealed that the distribution was not significantly different from a normal distribution after log-transformation, for Chinese participants *p* = .13; for German participants *p* = .82).

**Table 1:** Comparison pairs in the non-symbolic numerical magnitude comparison task. Each of the 16 comparison pairs contained a reference numerosity (either 16 or 32 dots) and a deviant numerosity (either between 12 and 20 or between 24 and 40 dots).

reference numerosity	deviant	ratio
16/32	12/24	0.750
16/32	13/26	0.8125
16/32	14/28	0.875
16/32	15/30	0.9375
16/32	17/34	1.0625
16/32	18/36	1.125
16/32	19/38	1.1875
16/32	20/40	1.25

#### Arithmetic

Simple arithmetic tasks were used to compare mathematical performance of both groups because solving these kinds of tasks is assumed to rely on the processing of numerical magnitudes (e.g., [8,24]). The whole set of problems consisted of four blocks of 110 arithmetical problems; two blocks of addition problems and two blocks of subtraction problems. The addition problems required adding two single-digit numbers (excluding 0 and 1) and were divided into one block without decade breaks (solutions ranging from 5 to 10) and another block with decade breaks (solutions ranging from 11 to 17). Ties (e.g., 4 + 4) were not included. The block without decade breaks consisted of 24 and the block with decade breaks consisted of 32 problems. The respective inverse tasks were used as subtraction problems (e.g., 5–3 and 5–2 as inverse tasks of 2 + 3 and 3 + 2). Within the different blocks, the problems were presented in pseudo-randomized order ensuring that neither identical nor commutated problems followed each other directly. The repetition rate of the different problems varied. The problems were presented in written form and the participants were asked to write down solutions for all problems. The sum of response times for the four blocks was used to estimate arithmetic performance and log-transformed to yield more normally distributed data (the Shapiro-Wilk test revealed that the distribution was not significantly different from a normal distribution after log-transformation; Chinese participants: *p* = .98; German participants: *p* = .20).

#### Reasoning

Raven’s Standard Progressive Matrices Plus (SPM Plus; [22]) were used to assess inductive reasoning. The SPM Plus is an untimed power test consisting of 60 non-colored diagrammatic puzzles, each with a missing part which has to be identified from a choice of six or eight options. Total scores ranging from 0 to 60 were used to estimate reasoning abilities.

#### Processing speed

A visual detection task was used to assess individual processing speed. Participants were instructed to press the space bar of the computer’s keyboard as soon as possible whenever an “X” appeared in the center of the screen. The target appeared until a response was given, but only up to a maximum duration of 3000 ms. The task comprised 60 experimental trials with varying inter-trial intervals (2000, 3500, 5000, 6500, or 8000 ms). Correct responses were used for computing mean RT. If no response was given, a trial was classified as erroneous.

#### Statistical analyses

The raw data is given in S1 Table. By using two-sample t-tests, Chinese and German participants were compared with regard to reasoning abilities, response times in the addition and subtraction tasks, as well as with regard to mean RT and mean ER in the non-symbolic numerical magnitude comparison task. Logistic regression models were used to compare age, mean ER in the addition and subtraction tasks, mean RT and mean ER in the visual detection task, and mean ER in size-controlled trials of the non-symbolic numerical magnitude comparison task because the assumption of normality was violated for these variables.

To assess effects of ratio between the two to-be-compared numerosities in the non-symbolic numerical magnitude comparison task, we averaged over the two different reference numerosity conditions and used polynomial linear trend analyses for deviants smaller than the reference and for deviants larger than the reference separately for Chinese and German participants.

We used mediation analyses in order to test whether a possible difference in performance between Chinese and German participants in the non-symbolic numerical magnitude comparison task was mediated by arithmetic skills. On the one hand, mediation analysis allows to investigate direct associations used in this study to examine the relation between the factor group (Chinese vs. German) and individual markers of the ANS, while holding constant the performance in the arithmetic tasks. On the other hand, mediation analysis provides estimates of the statistical significance of indirect associations, used in this study to evaluate whether arithmetic skills mediate the association between the factor group and individual markers of the ANS. In addition, mediation analysis allowed us to examine whether there is an association between individual markers of the ANS and arithmetic skills, while holding constant the factor group (Chinese vs. German). A second mediation model was tested to check the opposite direction of influence, i.e., to examine whether a possible performance difference between Chinese and German participants in the arithmetic tasks was mediated by the performance in the non-symbolic numerical magnitude comparison task. The mediation models were tested using the INDIRECT macro in SPSS [25]. This macro uses the bootstrapping method with bias-corrected confidence estimates. Confidence intervals (95%) for the indirect associations were obtained using 5000 bootstrap samples. If a confidence interval does not include zero, the indirect effect is deemed statistically different from zero representing evidence for a mediating effect [26].

## Results

Mean ER in the arithmetic tasks as well as in the visual detection task was low and did not significantly differ between groups (arithmetic: Chinese participants: .61%, SD .50, German participants: .74%, SD .71; Wald χ2 (1) = 1.47, *p* = .23, *odds ratio* = .71; visual detection: Chinese participants: .03%, SD .23, German participants: 0%, SD .00; Wald χ2 (1) = .00, *p* = 1.00) and was therefore not further analyzed. Moreover, Chinese and German participants did not differ with regard to age (Wald χ2 (1) = 1.28, *p* = .26, *odds ratio* = 1.13) and reasoning abilities (*t*(138) = .19, *p* = .85, *d* = .00). While German participants answered faster in the visual detection task (Wald χ2 (1) = 8.32, *p* = .004, *odds ratio* = 1.02), Chinese participants showed faster responses in the arithmetic tasks (log-transformed response times: *t*(138) = 10.98, *p* < .001, *d* = 1.87) and in the non-symbolic numerical magnitude comparison task (log-transformed RT: *t*(138) = 2.83, *p* = .005, *d* = .49; log-transformed RT in luminance-controlled trials: *t*(138) = 2.80, *p* = .006, *d* = .48; log-transformed RT in size-controlled trials: *t*(138) = 2.84, *p* = .005, *d* = .49). No significant differences were found for ER in the non-symbolic numerical magnitude comparison task (ER: *t*(138) = .31, *p* = .76, *d* = .00; ER in luminance-controlled trials: *t*(138) = .07, *p* = .95, *d* = .00; ER in size-controlled trials: Wald χ2 (1) = .46, *p* = .50, *odds ratio* = 6.34). See Table 2 for a depiction of these results.

**Table 2:** Comparison of Chinese and German participants.

	Chinese participants			German participants			*p* (two-sided)
	*M*	*SD*	*SE*	*M*	*SD*	*SE*	
age	20.8	1.6	.19	20.5	1.5	.18	*p* = .26
reasoning	45	5.3	.64	45	5.5	.66	*p* = .85
processing speed^a^	341	44.0	6.2	316	34.9	4.9	*p* = .004
arithmetic^b^	476	90.7	10.8	703	156.4	18.7	*p* < .001
RT comparison^b^	949	292.9	35.0	1105	346.5	41.4	*p* = .005
RT luminance-controlled^b^	996	326.6	39.0	1163	375.9	44.9	*p* = .006
RT size-controlled^b^	901	262.7	31.4	1046	321.0	38.4	*p* = .005
ER comparison	20	.06	.01	20	.07	.01	*p* = .76
ER luminance-controlled	25	.07	.01	25	.08	.01	*p* = .95
ER size-controlled	14	.06	.01	14	.06	.01	*p* = .50

Results of two-sample t-tests/logistic regression models comparing age, reasoning abilities, processing speed (in ms), response times (in s) in the arithmetic tasks as well as reaction times (in ms) and errors (in %) in the non-symbolic numerical magnitude comparison task (RT comparison, RT luminance-controlled, RT size-controlled, ER comparison, ER luminance-controlled, ER size-controlled) as well as means (*M*), standard deviations (*SD*) and standard errors of the mean (*SE*) separately for Chinese and German participants.

n = 140 (70 Chinese and 70 German participants);

^a^ n = 100 (50 Chinese and 50 German participants);

^b^ *p*-value based on analysis of log-transformed RT

**Fig 1:**
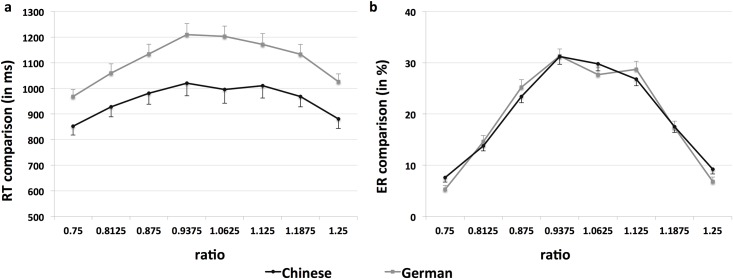
Performance in the non-symbolic numerical magnitude comparison task. Reaction times (in ms) and errors (in %) separately for Chinese and German participants as a function of the different ratios.

Demonstrating the signature of the ANS, performance in the non-symbolic numerical magnitude comparison task increased the more the ratio between the two to-be-compared numerosities deviated from one: significant linear trends for deviants smaller than the reference (ratios: .750 vs. .8125 vs. .875 vs. 9375; log-transformed RT: Chinese participants: *F*(1, 69) = 77.55, *p* < .001, η_p_^2^ = .53; German participants: *F*(1, 69) = 75.52, *p* < .001, η_p_^2^ = .52; ER: Chinese participants: *F*(1, 69) = 278.04, *p* < .001, η_p_^2^ = .80; German participants: *F*(1, 69) = 384.87, *p* < .001, η_p_^2^ = .85) and for deviants larger than the reference (ratios: 1.0625 vs. 1.125 vs. 1.1875 vs. 1.25; log-transformed RT: Chinese participants: *F*(1, 69) = 56.80, *p* < .001, η_p_^2^ = .45; German participants: *F*(1, 69) = 84.58, *p* < .001, η_p_^2^ = .55; ER: Chinese participants: *F*(1, 69) = 212.64, *p* < .001, η_p_^2^ = .76; German participants: *F*(1, 69) = 248.57, *p* < .001, η_p_^2^ = .78) were found in both groups (see Fig 1).

**Fig 2:**
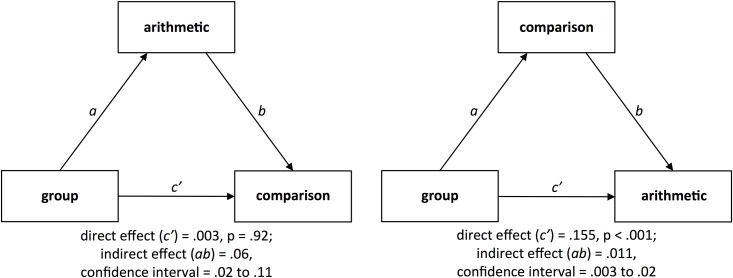
Mediation models. Left panel: Model testing whether log-transformed response times in the arithmetic tasks mediate the association between the factor group (Chinese vs. German) and log-transformed RT in the non-symbolic numerical magnitude comparison task. Right panel: Model testing whether log-transformed RT in the non-symbolic numerical magnitude comparison task mediate the association between the factor group (Chinese vs. German) and log-transformed response times in the arithmetic tasks.

The first mediation model revealed that the group difference in log-transformed RT in the non-symbolic numerical magnitude comparison task was no longer significant after controlling for log-transformed response times in the arithmetic tasks (direct effect = .003, *t*(138) = .11, *p* = .92) and it was significantly mediated by arithmetic performance (indirect effect = .06; confidence interval = .02 to .11; see Fig 2). Moreover, log-transformed RT in the non-symbolic numerical magnitude comparison task was found to be significantly associated with arithmetic skills even after controlling for group membership (*r* = .25, *p* = .004 [two-sided]). The second mediation model, by contrast, showed that the group difference in arithmetic performance was still significant after controlling for log-transformed RT in the non-symbolic numerical magnitude comparison task (direct effect = .16, *t*(138) = 10.27, *p* < .001). However, the group difference in arithmetic performance was significantly mediated by log-transformed RT in the non-symbolic numerical magnitude comparison task (indirect effect = .01; confidence interval = .003 to .02; see Fig 2). Similar results were found when reasoning or processing speed were used as control variables in the mediation models (see S1 Text).

Significant negative correlations between log-transformed RT and ER in the non-symbolic numerical magnitude comparison task were found in both groups (Chinese participants: r = -.53, p < .001 [two-sided]; German participants: r = -.52, p < .001 [two-sided]), representing evidence for a speed-accuracy trade-off. As a consequence, we calculated composite scores by z-transforming mean accuracy and log-transformed mean RT separately before averaging these two values (see e.g., [27]). Comparison of Chinese and German participants regarding these composite scores revealed a marginally significant difference (*t*(138) = 1.78, *p* = .078, *d* = .30). Moreover, using the composite scores instead of log-transformed RT in the mediation models did not change the results substantially: The first mediation model revealed that the group difference in the non-symbolic numerical magnitude comparison task was no longer (marginally) significant after controlling for log-transformed response times in the arithmetic tasks (direct effect = .056, *t*(138) = .28, *p* = .78) and it was significantly mediated by arithmetic performance (indirect effect = -.32; confidence interval = -.07 to -.64). Moreover, performance in the non-symbolic numerical magnitude comparison task was found to be significantly associated with arithmetic performance even after controlling for group membership (*r* = .20, *p* = .020 [two-sided]). The second mediation model, by contrast, showed that the group difference in arithmetic performance was still significant after controlling for performance in the non-symbolic numerical magnitude comparison task (direct effect = -.161, *t*(138) = -10.68, *p* < .001). However, the group difference in arithmetic performance was significantly mediated by performance in the non-symbolic numerical magnitude comparison task (indirect effect = -.005; confidence interval = -.0001 to -.0166).

## Discussion

We compared Chinese and German adults regarding their performance in arithmetic tasks and in a non-symbolic numerical magnitude comparison task. In line with previous findings, Chinese participants showed better performance in the arithmetic tasks (see e.g., [18,19,20,21]). They solved simple addition and subtraction problems significantly faster than German participants. This superior arithmetic performance of Chinese participants was not found to be accompanied by a more accurate performance in the non-symbolic numerical magnitude comparison task. Indeed, Chinese and German participants showed similar patterns of ER (see Fig 1b), suggesting that the average precision of non-symbolic numerical magnitude representations was comparable in both groups. Chinese participants were, however, overall faster in comparing dot arrays with respect to their quantity. Thus, Chinese adults not only showed a higher fluency in solving simple arithmetic tasks but were also able to discriminate between sets of different numerical quantities at a faster pace than their German peers. These performance differences cannot be ascribed to differences in general cognitive abilities. Chinese and German participants showed similar reasoning abilities, and the group difference in processing speed (German participants answered significantly faster than Chinese participants) did not converge with the group differences in the arithmetic tasks and in the non-symbolic numerical magnitude comparison task. Moreover, Chinese participants answered significantly faster than German participants in luminance-controlled and in size-controlled trials of the non-symbolic numerical magnitude comparison task, confirming that the visual characteristics of the stimuli did not differentially affect the performance of both groups.

As suggested by the present findings, a higher familiarity with arithmetic comes along with a higher speed of retrieving non-symbolic numerical magnitude knowledge. Previous findings by Guillaume and colleagues [14], however, revealed that adults with better arithmetic skills show more precise non-symbolic numerical magnitude representations. This might be related to the fact that response times in the non-symbolic numerical magnitude comparison task used by Guillaume and colleagues [14] were not restricted, while they were restricted to 4000 ms in the task we used. This restriction might have emphasized processing speed, and, indeed, mean correct RT in the present study was lower than reported by Guillaume and colleagues (2013; 1227 vs. 1027 ms) even though they used easier ratio conditions (0.5, 0.6, 0.67, 0.75, 0.89 [ER = 11%] vs. 0.750, 0.8125, 0.875, 0.9375 [ER = 20%] in the present study). Depending on the specific task demands, better arithmetic skills may therefore either be accompanied by more accurate or by faster responses in a non-symbolic numerical magnitude comparison task. As accuracy and speed in the non-symbolic numerical magnitude comparison task were found to be positively correlated in the present study, performance of Chinese and German participants was also compared on the basis of composite scores considering both accuracy and speed. This analysis revealed a marginally significant group difference. Thus, even when considering both accuracy and speed, there was still evidence for a group difference in processing of non-symbolic numerical magnitude.

Mediation analysis revealed that the group difference in non-symbolic numerical magnitude processing was fully mediated by the performance in arithmetic tasks. After controlling for arithmetic performance, the group difference in non-symbolic numerical magnitude processing was no longer significant. This was found to be the case regardless of whether RT or the composite scores considering both accuracy and speed in the non-symbolic numerical magnitude comparison task were used as dependent variable. These findings can be seen as evidence for the notion that arithmetic skills shape non-symbolic numerical magnitude processing skills. In this context, it has been suggested that the experience and familiarity with symbolic numerical information might be a key factor exerting influence on non-symbolic numerical magnitude processing skills [12]. Arithmetic problem solving is assumed to involve the retrieval of numerical magnitude knowledge [8], supposedly leading to activation of plausible answers and allowing for the detection of implausible ones [24]. Processing approximate numerical magnitude information thus seems to play an important role during arithmetic problem solving and it can be assumed that non-symbolic numerical magnitude processing skills are reciprocally related to arithmetic learning. Accordingly, higher experience and familiarity with arithmetic in Chinese compared to German adults may lead to better non-symbolic numerical magnitude processing skills. It can, however, not be ruled out that other factors are responsible for the mediation effect detected in the present study. For example, the more regular and transparent Chinese number word system may explain Chinese adults' advantage in the arithmetic tasks (see, e.g., [28,29]). If Chinese and German participants attempted to count the dots presented in the non-symbolic numerical magnitude comparison task, differences in the structure of the number naming systems may explain Chinese adults' advantage in this task. The mean response speed in the non-symbolic numerical magnitude comparison task (Chinese participants: 949 ms, German participants: 1105 ms), however, makes it unlikely that our participants attempted to count the presented dots. The mediation effect detected in the present study could also be based on better sensory integration skills (see [9]) of the Chinese adults in comparison to their German peers, which might have influenced performance in both the arithmetic tasks as well as the non-symbolic numerical magnitude comparison tasks. It was recently demonstrated that visual perception skills account for the association between non-symbolic numerical magnitude processing skills and arithmetic performance in Chinese children [30]. Future studies may thus examine the potential influence of perceptual skills on the association between non-symbolic numerical magnitude processing skills and math performance in greater depth.

Mediation analysis also revealed that the difference between Chinese and German participants in arithmetic performance was partially mediated by non-symbolic numerical magnitude processing skills. Indeed, the group difference in arithmetic performance was significantly mediated by the performance in the non-symbolic numerical magnitude comparison task but it was still significant after controlling for the performance in the non-symbolic numerical magnitude comparison task.

This was found to be the case regardless of whether RT or the composite scores considering both accuracy and speed in the non-symbolic numerical magnitude comparison task were used as mediating variable. Differences in non-symbolic numerical magnitude processing skills thus contribute to differences in arithmetic performance of young adults. Indeed, non-symbolic numerical magnitude processing skills seem to play a role in explaining the performance difference between Chinese and German adults in arithmetic tasks but we can expect the presence of more important explanatory factors, like the frequency of exposure to arithmetic, the structure of number naming systems as well as cultural beliefs and values (e.g., [28]).

While the group difference in arithmetic performance was only partially mediated by non-symbolic numerical magnitude processing skills, the group difference in non-symbolic numerical magnitude processing was fully mediated by the performance in arithmetic tasks. The influence of non-symbolic numerical magnitude processing skills on arithmetic skills accordingly seems to be lower than the opposite direction of influence, at least in a population where arithmetic processing is an accomplished skill. It is important to note, however, that the cross-sectional design of the current study does not offer means of assessing cause. Based on the different results of the two mediation models, we assume that a higher degree of familiarity with arithmetic in Chinese compared to non-Chinese adults causes better non-symbolic numerical magnitude processing skills. To substantiate this notion, longitudinal studies are needed. By assessing both the development of non-symbolic numerical magnitude processing skills and the development of arithmetic skills in Chinese and German participants over a long period of time, we would gain a better understanding of the interrelationship between these skills. Moreover, it would be possible to examine whether the direction of influence changes in the course of development and to determine to what extent the developmental trajectories are culture-specific.

To conclude, results from our study revealed that differences in arithmetic performance are accompanied by differences in processing of non-symbolic numerical magnitude. A higher familiarity with arithmetic was found to come along with an advantage in non-symbolic numerical magnitude processing. This advantage became evident by a higher speed of retrieving non-symbolic numerical magnitude knowledge but not by a higher precision of non-symbolic numerical magnitude representations. Differences in the speed of retrieving non-symbolic numerical magnitude knowledge were fully mediated by arithmetic performance, suggesting that arithmetic skills shape non-symbolic numerical magnitude processing skills.

## Supporting information

S1 Table Raw data. (XLSX)

S1 Text Results of mediation models with reasoning or processing speed as control variable. (DOCX)

## References

[1] Piazza M. Neurocognitive start-up tools for symbolic number representations. Trends Cogn Sci. 2010;14(12):542–51. 10.1016/j.tics.2010.09.008 21055996

[2] Van Oeffelen MP, Vos PG. A probabilistic model for the discrimination of visual number. Percept Psychophys. 1982;32:163–170. 7145586 10.3758/bf03204275

[3] Izard V, Sann C, Spelke ES, Streri A. Newborn infants perceive abstract numbers. PNAS. 2009;106:10382–10385. 10.1073/pnas.0812142106 19520833 PMC2700913

[4] Halberda J, Ly R, Wilmer JB, Naiman DQ, Germine L. Number sense across the lifespan as revealed by a massive Internet-based sample. PNAS. 2012;109(28):11116–20. 10.1073/pnas.1200196109 22733748 PMC3396479

[5] Piazza M, Pica P, Izard V, Spelke ES, Dehaene S. Education Enhances the Acuity of the Nonverbal Approximate Number System. Psychol Sci. 2013;24:1037–1043. 10.1177/0956797612464057 23625879 PMC4648254

[6] Chen Q, Li J. Association between individual differences in non-symbolic number acuity and math performance: A meta-analysis. Acta Psychol. 2014;148:163–172. 10.1016/j.actpsy.2014.01.016 24583622

[7] Fazio LK, Bailey DH, Thompson CA, Siegler RS. Relations of different types of numerical magnitude representations to each other and to mathematics achievement. J Exp Child Psychol. 2014;123(1):53–72. 24699178 10.1016/j.jecp.2014.01.013

[8] Schneider M, Beeres K, Coban L, Merz S, Schmidt SS, Stricker J, et al Associations of Non-Symbolic and Symbolic Numerical Magnitude Processing with Mathematical Competence: A Meta-analysis. Dev Sci. Forthcoming. 10.1111/desc.12372 26768176

[9] Gebuis T, Kadosh RC, Gevers W. Sensory-integration system rather than approximate number system underlies numerosity processing: A critical review. Acta Psychol. 2016;171:17–35. 10.1016/j.actpsy.2016.09.003 27640140

[10] Leibovich T, Katzin N, Harel M, Henik A. From ‘sense of number’ to ‘sense of magnitude’—The role of continuous magnitudes in numerical cognition. Behav Brain Sci. Forthcoming. 10.1017/S0140525X16000960 27530053

[11] Zebian S, Ansari D. Differences between literates and illiterates on symbolic but not nonsymbolic numerical magnitude processing. Psychon Bull Rev. 2012;19(1):93–100. 10.3758/s13423-011-0175-9 22033982

[12] Nys J, Ventura P, Fernandes T, Querido L, Leybaert J. Does math education modify the approximate number system? A comparison of schooled and unschooled adults. Trends Neurosci Educ. 2013;2,13–22.

[13] Castronovo J, Göbel SM. Impact of high mathematics education on the number sense. PLoS One. 2012;7(4):e33832 10.1371/journal.pone.0033832 22558077 PMC3338810

[14] Guillaume M, Nys J, Mussolin C, Content A. Differences in the acuity of the Approximate Number System in adults: The effect of mathematical ability. Acta Psychol. 2013;144(3):506–512. 10.1016/j.actpsy.2013.09.001 24096088

[15] Lindskog M, Winman A, Juslin P. The association between higher education and approximate number system acuity. Front Psychol. 2014;5:462 10.3389/fpsyg.2014.00462 24904478 PMC4033103

[16] Mullis IVS, Martin MO, Foy P, Arora A. TIMSS 2011 International Results in Mathematics. Chestnut Hill, MA: TIMSS & PIRLS International Study Center, Boston College; 2012.

[17] OECD. PISA 2012 Results: What Students Know and Can Do—Student Performance in Mathematics, Reading and Science (Volume I). PISA, OECD Publishing; 2013.

[18] Wang J, Lin E. A meta-analysis of comparative studies on Chinese and US students’ mathematics performance: implications for mathematics education reform and research. Educ Res Rev. 2009;4:177–195.

[19] Wang J, Lin E. Corrigendum to a meta-analysis of comparative studies on Chinese and US student mathematics performance: implications for mathematics education reform and research [Edu. Res. Rev. 4 (3)(2009) 177–195]. Educ Res Rev. 2013;10:150–157.

[20] Campbell JI, Xue Q. Cognitive arithmetic across cultures. J Exp Psychol Gen. 2001;130(2):299–315. 11409105 10.1037//0096-3445.130.2.299

[21] Geary DC, Salthouse TA, Chen G-P, Fan L. Are East Asian versus American differences in arithmetical ability a recent phenomenon? Dev Psychol. 1996;32:254–262.

[22] Horn R. Standard progressive matrices (SPM-C/SPM-P/SPM Plus). Frankfurt am Main: Pearson; 2009.

[23] Inglis M, Gilmore C. Indexing the approximate number system. Acta Psychol. 2014;145(1):147–155. 10.1016/j.actpsy.2013.11.009 24361686

[24] Siegler RS, Lortie-Forgues H. An integrative theory of numerical development. Child Dev. Perspect. 2014;8:144–150.

[25] Preacher KJ, Hayes AF. Asymptotic and resampling strategies for assessing and comparing indirect effects in multiple mediator models. Behav Res Methods. 2008;40(3):879–91. 18697684 10.3758/brm.40.3.879

[26] Hayes AF, Preacher KJ. Statistical mediation analysis with a multicategorical independent variable. Br J Math Stat Psychol. 2014;67:451–470. 10.1111/bmsp.12028 24188158

[27] Dietrich JF, Huber S, Klein E, Willmes K, Pixner S, Moeller K. A Systematic Investigation of Accuracy and Response Time Based Measures Used to Index ANS Acuity. PLoS ONE. 2016; 11:e0163076 10.1371/journal.pone.0163076 27637109 PMC5026358

[28] Ng SSN, Rao N. Chinese number words, culture, and mathematics learning. Rev Educ Res. 2010;80:180–206.

[29] Lonnemann J, Yan S. Does number word inversion affect arithmetic processes in adults? Trends Neurosci Educ. 2015;4:1–5.

[30] Zhou X, Wei W, Zhang Y, Cui J, Chen C. (2015). Visual perception can account for the close relation between numerosity processing and computational fluency. Front Psychol. 2015;6:1364 10.3389/fpsyg.2015.01364 26441740 PMC4563146

