# Supplementary material for: Differences in arithmetic performance between Chinese and German adults are accompanied by differences in processing of non-symbolic numerical magnitude
Source: PLoS One. 2017 Apr 6;12(4):e0174991. doi: 10.1371/journal.pone.0174991 (PMC5383159; doi:10.1371/journal.pone.0174991)
Supplement: S1 Text — (DOCX) [file pone.0174991.s002.docx]

**S1 Text. Results of mediation models with reasoning or processing speed as control variable.**

**Reasoning as control variable**

The first mediation model revealed that the group difference in log-transformed RT in the non-symbolic numerical magnitude comparison task was no longer significant after controlling for log-transformed response times in the arithmetic tasks (direct effect = .01, *t*(137) = .32, *p* = .75) and it was significantly mediated by arithmetic performance (indirect effect = .06; confidence interval = .01 to .11). Moreover, log-transformed RT in the non-symbolic numerical magnitude comparison task was found to be significantly associated with arithmetic skills even after controlling for group membership (*r* = .21, *p* = .013 [two-sided]). The second mediation model, by contrast, showed that the group difference in arithmetic performance was still significant after controlling for log-transformed RT in the non-symbolic numerical magnitude comparison task (direct effect = .16, *t*(137) = 10.61, *p* < .001). However, the group difference in arithmetic performance was significantly mediated by log-transformed RT in the non-symbolic numerical magnitude comparison task (indirect effect = .01; confidence interval = .002 to .02).

**Processing speed as control variable**

The first mediation model revealed that the group difference in log-transformed RT in the non-symbolic numerical magnitude comparison task was no longer significant after controlling for log-transformed response times in the arithmetic tasks (direct effect = .02, *t*(97) = .59, *p* = .56) and it was significantly mediated by arithmetic performance (indirect effect = .04; confidence interval = .002 to .09). Moreover, log-transformed RT in the non-symbolic numerical magnitude comparison task was found to be significantly associated with arithmetic skills even after controlling for group membership (*r* = .20, *p* = .045 [two-sided]). The second mediation model, by contrast, showed that the group difference in arithmetic performance was still significant after controlling for log-transformed RT in the non-symbolic numerical magnitude comparison task (direct effect = .13, *t*(97) = 6.78, *p* < .001). However, the group difference in arithmetic performance was significantly mediated by log-transformed RT in the non-symbolic numerical magnitude comparison task (indirect effect = .01; confidence interval = .0004 to .02).
